# Dr. H. W. Caldwell

**Published:** 1915-04

**Authors:** 


**Dr. H. W. Caldwell,** Buffalo, 1866, died at his home in Pulaski, Oswego County, February 25, aged 73, where he had practiced since 1872. After beginning the study of medicine, he enlisted in the U. S. Volunteers and was severely wounded in the battle of Wilmington Island, Ga., April 1862. After his recovery, he served as Hospital Steward till the winter of 1863, being attached to the 8th Michigan Infantry. He was coroner of Oswego County, 1872 to 1885.

